# Hereditary cancer syndromes: utilizing DNA repair deficiency as therapeutic target

**DOI:** 10.1007/s10689-016-9883-7

**Published:** 2016-02-12

**Authors:** Gaurav Goyal, Tiffany Fan, Peter Todd Silberstein

**Affiliations:** Department of Internal Medicine, CHI Health Creighton University Medical Center, Omaha, NE USA; Class of 2017, Creighton University School of Medicine, Omaha, NE USA; Division of Hematology/Oncology, CHI Health Creighton University Medical Center and VA Nebraska-Western Iowa Health Care System, Omaha, NE USA

**Keywords:** Hereditary cancer, DNA repair, BRCA, Lynch syndrome, Microsatellite high, PARP

## Abstract

Human cells have numerous repair mechanisms to counteract various insults incurred on the DNA. Any mutation in these repair mechanisms can lead to accumulation of DNA errors and carcinogenesis. This review aims to discuss the therapeutic options in the two most common DNA repair deficient cancer syndromes, namely Lynch syndrome (hereditary non-polyposis colorectal cancer) and breast cancer susceptibility gene (BRCA) associated ovarian and breast cancer. Deficiency in DNA repair mechanisms renders these tumors with increased sensitivity to platinum agents. There has been increasing amount of information on the utility of the defects in DNA repair as targets for cancer therapy in these syndromes. Novel therapies like poly (ADP-ribose) polymerase (PARP) inhibitors are one of such example where the induction of double stranded breaks in DNA leads to tumoricidal effect in patients with homologous DNA repair deficiency. Interestingly, patients with DNA repair deficiencies tend to have a more favorable prognosis than sporadic malignancies. In microsatellite high colorectal cancer patients, this has been attributed to increased recruitment of CD8+ T lymphocytes in tumor microenvironment. However, these tumors are able to limit the host immune response by activation of immune checkpoints that seem like attractive targets of therapy in the future.

## DNA repair mechanisms

Human cells are continuously exposed to countless insults, ranging from ultravoilet light and ionizing radiation to the use of alkylating and anti-tumor agents. In order to repair the harmful DNA damages that ensue, the human body is equipped with an intricate, interwoven damage control network comprised of five DNA repair mechanisms: base excision repair, mismatch repair, nucleotide excision repair, homologous recombination and non-homologous end-joining. Specifically, base excision repair is used to fix single stranded breaks and small base changes. Mismatch repair is used to correct A–G and T–C mismatches as well as insertions and deletions. Nucleotide excision repair is used to remove bulky adducts and intrastrand crosslinks. Homologous recombination and non-homologous end joining are used to fix double stranded breaks and remove interstrand crosslinks. Mutations in the genetic makeup of any of these mechanisms may result in defective DNA repair, potentially leading to an abnormal pathology [1]. This review article will discuss the latest therapeutic options in the major DNA repair deficient inherited cancer syndromes, including hereditary non-polyposis colorectal cancer (Lynch syndrome), and breast cancer susceptibility gene (BRCA) associated ovarian and breast cancer.

## Lynch syndrome (hereditary non-polyposis colorectal cancer)

DNA mismatch repair is a highly conserved mechanism primarily used to correct mismatched base pairs that arise as a result of replication errors or cellular damage [2]. It is composed of four main genes—*MLH1*, *MSH2*, *MSH6*, *PMS2*—that encode the mismatch repair (MMR) proteins necessary for identification and repair of mismatched bases. These proteins work in unison as two heterodimeric complexes: *MLH1/PMS2* and *MSH2*/*MSH6* [3]. The genes responsible for the stability of their respective heterodimeric partners are *MLH1* and *MSH2* [4]. When a defect in this proofreading system occurs, the loss of MMR protein results in an accumulation of errors within DNA microsatellite regions. This phenomenon is known as microsatellite instability (MSI) [5].

**Fig. 1:**
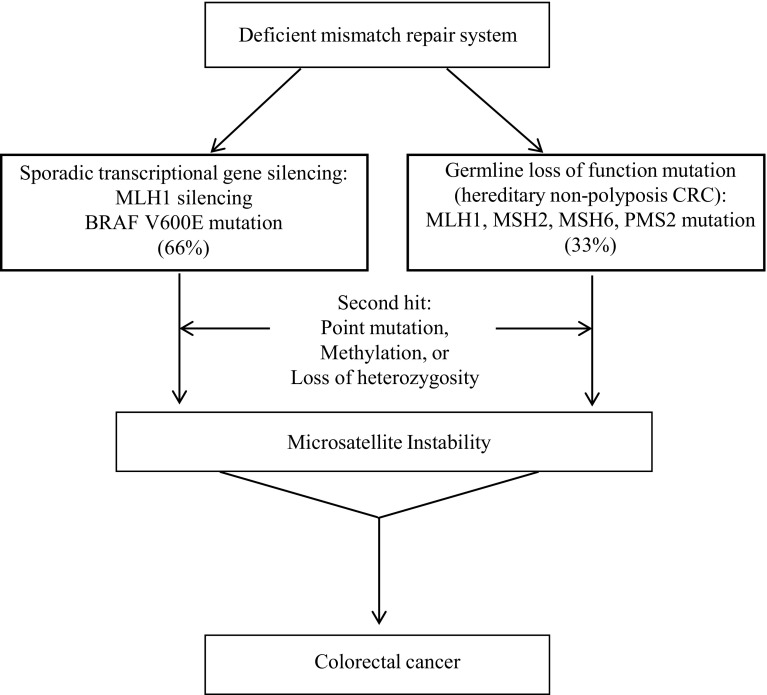
Molecular pathways for microsatellite instability (MSI) high colorectal cancer. About two-thirds of the cases are sporadic and involve transcriptional silencing of MLH1 gene that is always accompanied by a BRAF V600E mutation due to tight promoter correlation. The remaining one-third cases involve germline loss-of-function mutations in one of the mismatch repair (MMR) genes

Deficient mismatch repair causing microsatellite instability is responsible for 12–15 % of all colorectal cancers. Among this group, two-thirds are due to sporadic transcriptional gene silencing while the remaining third is due to a germline loss-of-function mutation [6]. In the sporadic pathway, hypermethylation of CpG islands in the promoter region causes MLH1 gene silencing to occur [2] (Fig. 1). This is always accompanied by a BRAF V600E mutation due to tight promoter correlation. Thus, MLH1 methylation and tumor BRAF mutations are indicative of negative DNA mismatch repair germline mutation status [7].

In contrast, deficient mismatch repair from a germline loss-of-function mutation is associated with Lynch Syndrome, an autosomal dominant syndrome formerly known as hereditary non-polyposis colorectal cancer. According to the International Society for Gastrointestinal Hereditary Tumors database, mutations in MLH1, MSH2, MSH6 and PMS2 account for 42, 33, 18 and 7 % of Lynch syndrome, respectively [8]. Pathology associated with Lynch Syndrome occurs only after a second hit, due to a somatic event such as a point mutation or methylation, damages the unaffected allele [3]. An alternative etiology for this syndrome is the germline epimutation of MLH1, a reversible hypermethylation event that involves various normal tissues [9]. In another subset of Lynch Syndrome patients, constitutional, biallelic 3′ exon deletion of the epithelial cell adhesion molecule can cause epigenetic silencing of the MSH2 gene and subsequent lack of MMR protein [10].

While cancer risks are elevated with the loss of any MMR protein [11], the risks are stratified, as cancer risks associated with MLH1 and MSH2 mutations are higher than with MSH6 and PMS2 mutations [12, 13]. The tumor spectrum in Lynch Syndrome is broad, with following cancers listed in order of decreasing frequency: colorectal, endometrial, gastric, biliopancreatic, and uroepithelial [14]. Thus, diligent screening is essential to decrease morbidity and mortality of patients with Lynch Syndrome [9].

Despite the increased risk of cancer, high-frequency microsatellite instability is associated with more favorable outcomes in colorectal cancer, i.e. lower stage of cancer and lower pathological stage [15] as well as a decreased likelihood of metastasis [16]. Data published on the prognosis of colorectal cancer in patients with a defect in mismatch repair support the notion that this deficiency is a positive prognostic factor in Stage II and III colorectal carcinoma [17, 18]. It is also associated with a lower recurrence rate of 11 % compared to 26 % in stage II and III colorectal cancer with an intact repair system [19]. Due to the inability to repair errors in DNA coding sequences, an accumulation of somatic mutations occurs, leading to the synthesis of neoantigens that are recognized by the body’s own immune system [20]. The immunogenicity of these neoantigens peptides creates a cytokine rich microenvironment with a high density of tumor-infiltrating lymphocytes, especially CD8+ T lymphocytes, that perhaps leads to the enhanced control over tumor growth and spread [21, 22]. While the prevalence of advanced, metastatic carcinoma is lower in mismatch repair deficient individuals [16], prognosis is poor at this stage, with lower disease free survival and overall survival than earlier stages [23]. The prognostic impact of DNA mismatch repair deficient individuals also depends on tumor site; proximal tumors have favorable outcomes and distal or N2 tumors correlate with worse outcomes [24].

Tumors with mismatch repair deficiency, are resistant to therapy with 5-Fluorouracil alone [25, 26]. However, available data suggests that mismatch repair deficient tumors are sensitive to platinum agents like oxaliplatin or FOLFOX (a combination of fluoropyrimidine, folic acid and oxaliplatin), as the sensitivity is independent of the repair system [27, 28]. The role of adjuvant FOLFOX in MMR deficient colorectal cancer patients was demonstrated by the long-term results from the MOSAIC (Multicenter International Study of Oxaliplatin/Fluorouracil/Leucovorin in the Adjuvant Treatment of Colon Cancer) study [29]. The study showed that MMR proficiency was an independent poor prognostic factor in colorectal cancer patients. However, the hazard ratio (HR) benefit for DFS and OS in FOLFOX4 [bolus/infusional fluorouracil plus leucovorin (LV5FU2) plus oxaliplatin] arm were 0.48 (95 % CI 0.20–1.12) and 0.41 (95 % CI 0.16–1.07), respectively, for stage II and III MMR-deficient patients, as compared to LV5FU2 arm [29]. This confirms a beneficial role of using FOLFOX in patients with stage III dMMR colorectal cancer.

Due to the resistance to 5-Fluorouracil, efforts are underway to identify novel therapies that exploit the DNA mismatch repair deficiency in microsatellite unstable colorectal cancers. A recent study conducted by Maby et al. [30] suggests that CD8+ tumor infiltrating lymphocyte density can be positively correlated with the total number of frameshift mutations, especially within the ASTE1, HNF1A and TCF7L2 genes. Studies examining the tumor microenvironment have shown that MSI tumors selectively increase the upregulation of immune checkpoint ligands such as PD-1 (programmed cell death 1), CTLA-4 (cytotoxic T-lymphocyte-associated protein 4), LAG-3 (lymphocyte-activation gene 3) and IDO (indoleamine 2,3-dioxygenase pathway), thereby preventing natural elimination of the tumor [31]. These findings have inspired research to design novel immunomodulatory therapy targeting this negative feedback system.

There are currently two classes of immunomodulatory monoclonal antibodies that are being developed to target tumors- cytotoxic T-lymphocyte-associated protein 4 (CTLA-4) and programmed death-1 (PD-1) antibodies. The anti CTLA-4 antibodies targets immunosuppressive receptors on the surfaces of T lymphocytes to overcome the effect of immune checkpoints [32]. The PD-1 inhibitors block the interaction of PD-1 molecule with programmed death-1 ligand (PD-L1) and facilitate tumor killing by activated T cells [32]. Currently, three antibodies are approved for oncological use: ipilimumab, nivolumab and pembrolizumab. Ipilimumab, a CTLA-4 inhibitor, was approved in 2011 for the treatment of metastatic melanoma. Nivolumab and pembrolizumab, programmed death-1 (PD-1) inhibitors, were approved in 2014 for cases of metastatic melanoma that progress on ipilimumab [32]. Currently, these agents have also been approved for use in advanced non-small cell lung carcinoma (both nivolumab and pembrolizumab) and renal cell carcinoma (nivolumab). The adverse effect profile of the anti PD-1 drugs seems to be less toxic than anti CTLA-4 antibody and includes fatigue, pruritus and rash [32].

A recent phase 2 study conducted by Le et al. [33] evaluated pembrolizumab in 41 patients with progressive metastatic carcinoma with or without mismatch-repair deficiency. Pembrolizumab was administered intravenously at a dose of 10 mg/kg every 2 weeks to both colorectal (n = 32) and non-colorectal (n = 9) cancer patients. All of the colorectal cancer patients except one had received two or more previous chemotherapy regimens and had similar duration of metastatic disease.

In the cohort with mismatch repair-deficient colorectal cancer patients (n = 11), the immune-related objective response rate (ORR) and immune-related progression free survival (PFS) rate at 20 weeks with pembrolizumab was 40 and 78 %, respectively. On the other hand, the ORR and PFS rate was 0 and 11 % for mismatch repair-proficient colorectal cancers (n = 21), thereby suggesting a clinical benefit with immune checkpoint blockade in patients with mismatch repair-deficient colorectal cancers [33]. Immunohistochemical analysis of the mismatch-repair deficient tumors showed a higher density of CD8-positive lymphoid cells and programmed death-1 ligand expression (PD-L1) as compared to the mismatch-repair proficient tumors. Of note, this study included a third cohort of mismatch-repair deficient non-colorectal cancer patients (n = 9) that showed an ORR of 71 % and 20-week PFS rate of 67 % [33].

Current research is also targeting synthetic lethal interactions of the mismatch repair pathway as well as secondary mutations [34]. Synthetic lethality between two genes is the concept that loss of function in one of the genes still produces a viable cell but loss of both genes results in cell death [35]. With regards to the mismatch repair pathway, there is evidence that inhibition of specific DNA polymerases in the base excision repair pathway is synthetically lethal with deficient mismatch repair proteins [34]. Specifically, nuclear base excision repair DNA polymerase ß is linked to MSH2 and mitochondrial DNA polymerase Υ is linked to MLH1 in such a manner that inhibition of the polymerase can induce an accumulation of oxidative DNA lesions in a mismatch repair deficient tumor [36]. The use of methotrexate, a folate antimetabolite, leads to selective accumulation of oxidative DNA lesions in MSH2 deficient cells due to their inability to clear the damage [37]. In light of this phenomenon, a phase II clinical trial testing the cytotoxic effects of methotrexate in MSH2 deficient colorectal cancer is underway (MESH, NCT00952016) [34]. In addition to this, secondary mutations are also a source that can be targeted. Studies have shown that secondary mutations in double stranded base repair genes are associated with primary mismatch repair gene mutations [38]. With synthetic lethality between double stranded base repair genes and poly (ADP-ribose) polymerase (PARP), a base excision repair enzyme, therapies combining methotrexate and a PARP inhibitor would allow for the accumulation of oxidative stress in a mismatch repair deficient tumor without the ability for DNA repair [34, 39].

## BRCA related ovarian cancer

BRCA 1 and BRCA 2 are two highly penetrant genes crucial to DNA damage repair and genomic stability [40]. By participating in the repair of double stranded breaks, they prevent the accumulation of gross chromosomal rearrangements that would ultimately lead to tumor formation [41]. BRCA1/2 proteins are particularly active agents in the error-free homologous recombination repair process [42, 43]. BRCA 1 is part of the BRCA1-associated genome surveillance complex (BASC) involved in the recognition and repair of aberrant DNA structures. The complex interacts with the MRE1/RA50/Nbs1 complex to reset double stranded break ends for homologous recombination [44], complexes with SWI/SNF for chromatin remodeling [45] and exhibits ubiquitin ligase activity [46]. BRCA 2 regulates RAD51 recombinase, a molecule that initiates ssDNA pairing during homologous recombination [47]. Inherited pathologic mutations in either BRCA genes destabilize the genome, predisposing the individual to a multitude of cancers.

Interestingly, BRCA-associated ovarian cancer has better prognosis than sporadic ovarian carcinoma [40]. Genomic instability from BRCA mutations sensitizes tumor cells to chemotherapeutic agents such as platinum salts, decreasing the mortality rate by 28 % [48]. In addition, patients experience longer disease-free intervals after primary chemotherapy as well as longer overall survival [49]. A retrospective cohort study of 933 ovarian cancers by Boyd et al. [49] showed that the group of BRCA-deficient ovarian cancers (stage III and IV) had improved survival and a longer disease-free interval following primary chemotherapy compared to the group of non-hereditary ovarian cancers. This phenomenon can be explained by the heightened sensitivity to platinum gents associated with the loss of BRCA proteins, conferring a greater response to chemotherapeutic agents. In cumulative survival analysis by subtype of BRCA mutation, patients with BRCA1 mutation had significantly longer survival than sporadic cases (*P* = 0.008), but BRCA2- linked cases only displayed a trend toward prolonger survival (*P* = 0.09).

One of the novel therapies entering phase II and III clinical trials is a poly (ADP-ribose) polymerase (PARP) inhibitor. PARP is an enzyme required for base excision repair [50]. When activated by a single strand break, PARP recruits DNA damage repair proteins to the site and facilitates the formation of a relaxed chromatin state to allow for DNA repair [51]. The effects of PARP inhibition are two-fold: single strand break repair complexes cannot be recruited to the site and PARPs that are already recruited to the site cannot undergo dissociation [52, 53]. This leads to replication fork stalling and eventual collapse with formation of double stranded DNA breaks [50, 53]. Without the ability for high fidelity homologous recombination to repair the breaks in BRCA mutant cases, an accumulation of DNA damage occurs and cellular apoptosis ensues [50]. It is the discovery of synthetic lethality between PARP and homologous recombination repair that has launched the use of PARP inhibitors for the treatment of several cancers.

Currently, PARP inhibitors, such as olaparib, are undergoing clinical development to target a wide variety of cancer types, including BRCA mutated breast and ovarian cancers [54–57]. The United States Food and Administration has also approved olaparib for use in BRCA-mutated ovarian cancers resistant to three prior chemotherapy regimens [58]. The current recommendation is treatment with 400 mg of olaparib twice a day beginning no later than 8 weeks after neoadjuvant platinum chemotherapy [59]. This was based on a phase II trial of 193 patients with advanced ovarian cancer who had a germline BRCA1/2 mutation. All of the patients received prior therapy with platinum agents and were considered to be platinum resistant. Olaparib use was associated with a tumor response rate of 31 % with a complete response seen in 3 % of the cases. Partial responses were seen in 28 % of the patients and stable disease ≥8 weeks was observed in 40 % of the patients with ovarian cancer. [59] Common side effects of PARP inhibitors include fatigue, nausea, vomiting, diarrhea, anorexia and dizziness. More serious adverse effects include hematological toxicities, myelodysplastic syndrome, acute myeloid leukemia and pneumonitis [60].

## BRCA related breast cancer

While there is no standard chemotherapy regimen to treat BRCA 1 or 2 mutated breast cancers, which are typically high grade and triple negative in nature, clinical trials have shown the superior efficacy of platinum salts as part of neoadjuvant chemotherapy to shrink the tumor prior to surgery [61]. Cisplatin and other platinum agents have the ability to crosslink and damage DNA strands that can only be repaired by high-fidelity homologous recombination typically absent in BRCA mutated cells [62]. Without BRCA proteins, there is a fivefold reduction in DNA double-stranded break repair via homologous recombination [43]. BRCA-deficient cells have amplified radiation sensitivity and a greater cellular response to ionizing radiation [63], although one might hypothesize that this might make them more susceptible to radiation induced secondary malignancies.

A large observational study by Byrski et al. [64] estimated the rates of pathologic complete response (pCR) for various neoadjuvant chemotherapy regimens given to young women with BRCA-1 positive breast cancers. Of the 102 women who carried a BRCA1 mutation, 24 patients were able to reach pCR. Among the 12 patients that received cisplatin, 83 % (n = 10) were able to achieve a pCR. A pCR of 7 % was observed for those treated with cyclophosphamide, methotrexate and fluorouracil (CMF), 8 % for those treated with doxorubicin and docetaxel (AT) and 22 % for those treated with doxorubicin and cyclophosphamide with and without fluorouracil (FAC, AC) [64].

The same research group recently completed a study to further evaluate the use of platinum-based neoadjuvant chemotherapy for women with triple-negative, BRCA1 mutated breast cancer [65]. Cisplatin was administered at a dose of 75 mg/m^2^ every 3 weeks for 4 cycles to 107 women diagnosed with stage I to III breast cancer, followed by surgery and conventional chemotherapy. Ninety-three of the patients had primary breast cancer and 14 of the patients were previously treated for cancer. After cisplatin chemotherapy, 65 out of the 107 patients (61 %) had achieved pCR. Further analysis determined that the rate of pCR was 56 % for women with ER-positive breast cancer, 61 % for those with triple-negative breast cancer, 73 % for those with node-negative cancer and 48 % for those with node-positive breast cancer. Since high pCR is suggestive of greater recurrence-free survival, this data suggests that platinum-based agents may be an effective neoadjuvant chemotherapeutic option for women with BRCA-1 positive breast cancer [65].

A recent randomized phase III trial by Tutt et al. evaluated the use of carboplatin compared with docetaxel in 376 patients with metastatic or recurrent locally advanced triple negative or BRCA-mutated breast cancer. Patients were randomized to either the carboplatin arm or the docetaxel arm and were treated for 6–8 cycles or until disease progression if sooner. At the conclusion of the study, the objective response rate (ORR) for the 43 BRCA-mutated breast cancer patients was 68.0 % with carboplatin compared to 33.3 % with docetaxel (*P* = 0.03). There was a no significant difference for non-BRCA patients, with an ORR of 28.1 versus 36.6 % for carboplatin and docetaxel, respectively (*P* = 0.16) [66].

Although platinum drugs are often used as monotherapy in individuals with BRCA1 mutations, studies show that combining platinum-based agents and conventional chemotherapy can also achieve a high pathologic complete response [67–69]. A recently published prospective study demonstrated that neoadjuvant carboplatin/docetaxel chemotherapy allowed for a pCR of 86 % in BRCA-associated triple negative breast cancers compared to a pCR of 50 % in sporadic, non-BRCA associated triple negative breast cancers [70]. Similarly, in another randomized phase II trial, carboplatin with weekly paclitaxel/doxorubicin was able to achieve a pCR of 53.2 %, compared to 36.7 % without the carboplatin [67]. Despite the success of platinum drugs, resistance to these agents have led to innovative research to discover biologic agents that target other repair mechanisms with synthetic lethality towards homologous recombination.

A proof-of-concept phase II trial engineered by Tutt et al. evaluated the use of olaparib in 54 patients with confirmed recurrent, advanced BRCA1 or BRCA 2 mutated breast cancer. Patients were either given continuous oral olaparib at the maximum tolerated dose of 400 mg twice daily (n = 27) or a low dose of 100 mg twice daily (n = 27). This study revealed a therapeutic objective response rate of 41 % in patients assigned to 400 mg twice daily compared to 22 % in patients assigned to 100 mg twice daily [60]. Further, the combination of olaparib with cisplatin has also proven efficacious, with a therapeutic response rate of 73 % in BRCA related breast cancers [54]. Other combinations with PARP inhibitors include neoadjuvant chemotherapy with carboplatin or topotecan [71–73]. There also seems to be some synergistic activity between veliparib, another PARP inhibitor, and temozolomide, with a clinical benefit rate of 50 % [74]. Apart from olaparib and veliparib, three other PARP inhibitors—niraparib, talazoparib and rucaparib—are also undergoing current investigation for use in advanced settings in germline BRCA mutated breast cancer [75].

Hence we can see how the landscape of therapeutics in DNA repair deficient cancer syndromes has evolved from traditional chemotherapy towards targeted novel therapies that aim to prolong survival with less toxicity. The use of platinum agents in these syndromes has already led to improved survival. With an increasing understanding of DNA repair defects, the DNA damage repair (DDR) agent olaparib is probably just a beginning of the utilization of synthetic lethality in treatment of hereditary cancers. Immune checkpoint inhibitors also seem to be a promising avenue in the management of hereditary cancers that evade the immune system by limiting the cytotoxic T cell response to these tumors. As more data becomes available on genetic testing, we might be able to have more information on expanding its scope to include other malignancies. One might be tempted to hypothesize that increased knowledge of DNA repair deficiencies could perhaps open doors to the use of targeted therapy in metastatic cancers of unknown primary that possess such defects.
